# An Update on Innate Immune Responses during SARS-CoV-2 Infection

**DOI:** 10.3390/v13102060

**Published:** 2021-10-14

**Authors:** Yu Zhang, Shuaiyin Chen, Yuefei Jin, Wangquan Ji, Weiguo Zhang, Guangcai Duan

**Affiliations:** 1 Department of Epidemiology, College of Public Health, Zhengzhou University, Zhengzhou 450001, China; 202022272014965@gs.zzu.edu.cn (Y.Z.); sychen@zzu.edu.cn (S.C.); ayq116814@gs.zzu.edu.cn (W.J.); wzhang033@icloud.com (W.Z.); gcduan@zzu.edu.cn (G.D.); 2 Suzhou Institute of Systems Medicine, Chinese Academy of Medical Sciences, Suzhou 215123, China

**Keywords:** SARS-CoV-2, innate immune response, type I interferons

## Abstract

The severe acute respiratory syndrome coronavirus 2 (SARS-CoV-2) is a member of the *Coronaviridae* family, which is responsible for the COVID-19 pandemic followed by unprecedented global societal and economic disruptive impact. The innate immune system is the body’s first line of defense against invading pathogens and is induced by a variety of cellular receptors that sense viral components. However, various strategies are exploited by SARS-CoV-2 to disrupt the antiviral innate immune responses. Innate immune dysfunction is characterized by the weak generation of type I interferons (IFNs) and the hypersecretion of pro-inflammatory cytokines, leading to mortality and organ injury in patients with COVID-19. This review summarizes the existing understanding of the mutual effects between SARS-CoV-2 and the type I IFN (IFN-α/β) responses, emphasizing the relationship between host innate immune signaling and viral proteases with an insight on tackling potential therapeutic targets.

## 1. Introduction

At the end of 2019, a pneumonia outbreak associated with a novel coronavirus (CoV) was reported [1]. The clinical manifestations of patients included fever, cough, pyrexia, acute respiratory distress, lymphopenia, and so on [2]. This emerging infectious disease was caused by severe acute respiratory syndrome coronavirus 2 (SARS-CoV-2) [3], and the World Health Organization (WHO) subsequently announced the name “Coronavirus Disease 2019” (COVID-19) for the novel pneumonia [4]. COVID-19 has the characteristics of high infectivity, fast transmission, long incubation period, and high concealment [5], which has made it rapidly spread all over the world [6]. As of 12 October 2021, SARS-CoV-2 infection has resulted in more than 238 million confirmed cases and more than 4,856,000 deaths [7]. This unexpected pandemic caused by SARS-CoV-2 has dealt a major blow to healthcare and economic systems across the world.

The immune system of the body protects us against various pathogens to keep healthy [8]. As we all know, the immune system consists of two parts called the innate (non-specific) and the adaptive (specific) immune system. The innate immune system is the body’s first line of defense against invading pathogens, and it can be activated by the identification of harmful factors. Harmful factors can be recognized through germline-encoded pattern recognition receptors (PRRs), and PRRs detect pathogen-associated molecular patterns (PAMPs) and damage-associated molecular patterns (DAMPs). The activated PRRs will quickly induce the host’s various defense programs to stop harmful invaders such as viruses. However, SARS-CoV-2 has diverse strategies to suppress the response to the innate immune system.

The disorder of the innate immune responses often leads to the occurrence of various diseases [9]. The dysfunction of innate immune responses, characterized by the weak generation of type I interferons (IFNs) and the hypersecretion of pro-inflammatory cytokines, results in the mortality and organ injury in patients with COVID-19. The cellular signaling induced by SARS-CoV-2 can not only offer useful information for uncovering mechanisms underlying the complicated outcomes of COVID-19 but also assist in devising treatment strategies for SARS-CoV-2 infection. Our review mainly focused on the existing understanding of the mutual effects between SARS-CoV-2 and the type I IFN response, emphasizing the relationship between host innate immune signaling and viral proteases with an insight on tackling potential therapeutic targets.

## 2. SARS-CoV-2 Genome, Protein and Its Host Receptors

SARS-CoV-2 was firstly isolated from the bronchoalveolar lavage fluid (BALF) that was collected from the COVID-19 patients of Jinyintan Hospital in Wuhan on 30 December 2019 [10]. SARS-CoV-2, like SARS-CoV and the Middle East respiratory syndrome CoV (MERS-CoV), belongs to the beta-CoVs (β-CoVs) and is considered primarily a bat-derived CoV [11]. SARS-CoV-2, a spherical or pleomorphic coated particle, consists of single-stranded RNA (ssRNA) associated with a nucleoprotein (N) in a capsid composed of matrix protein [12]. Similar to other β-CoVs, SARS-CoV-2 genome includes two untranslated regions (UTRs) and a big open reading frame (ORF) [13]. The SARS-CoV-2 genome is arranged in the 5′–3′ order as replicase (ORF1a/b)-Spike (S)-Envelope (E)-Membrane (M)-Nucleocapsid [14]. The SARS-CoV-2 genome contains about 30,000 nucleotides, encoding about 10,000 amino acids, and the G + C content is 38% [15]. The 5′-end ORF1a/b accounts for approximately two-thirds of the genome of SARS-CoV-2 and are translated to the large replicase polyprotein 1a (pp1a) and pp1ab [16]. These polyproteins are processed into 16 non-structural proteins (nsps, nsp1 to nsp16) by papain-like protease (PLpro), main-protease (Mpro) or 3C cleavage-like protease (3CLpro). The nsps combine to form the replication–transcription complex (RTC) that is made up of various enzymes, including RNA-dependent RNA polymerase (RdRp) and helicase (Hel). The RTC is involved in SARS-CoV-2 transcription and replication [17]. Near the 3′-terminus, one-third of the genome involves four structural proteins (S, N, E, M) and some accessory proteins such as ORF3a, ORF3b, ORF6 and so on [18,19]. The N protein wraps the genome of the virus, along with three other proteins to form the viral envelope [20]. The S protein is made up of two functional subunits, S1 and S2 [21].

**Figure 1:**
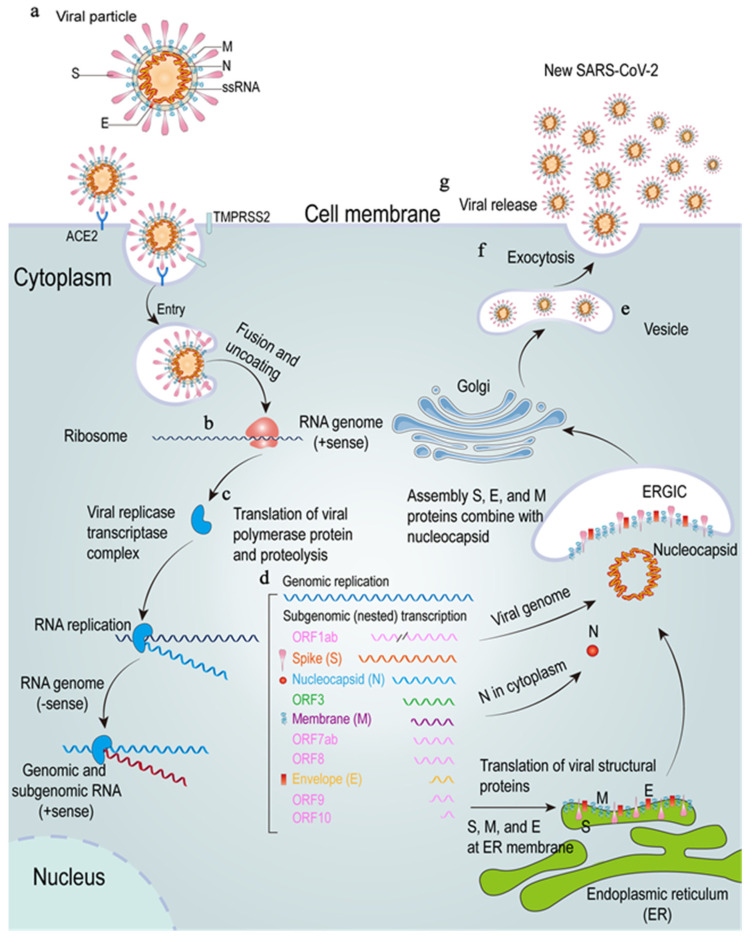
The SARS-CoV-2 lifecycle. (**a**–**d**), SARS-CoV-2 enters through membrane fusion and uncoating. The SARS-CoV-2 S protein combines with the ACE2 and TMPRSS2 to release RNA (**b**), and the genome RNA is translated into viral proteins (**c**). (**d**–**g**), these proteins will produce a viral replicase transcriptase complex to produce additional RNA (**d**). Subgenomic RNA transcription leads to the production of structural proteins and accessory proteins, which are translocated to endoplasmic reticulum (ER) membranes and go through the ER-Golgi intermediate compartment (ERGIC) to assemble a virus to produce new SARS-CoV-2 (**e**) and then will be released by exocytosis (**f**,**g**). ssRNA: single-stranded RNA; ACE2: angiotensin-converting enzyme 2; TMPRSS2: transmembrane serine protease 2; ORF1ab: open reading frame 1ab; ER: endoplasmic reticulum; ERGIC: ER-Golgi intermediate compartment.

SARS-CoV-2 mainly uses the transmembrane serine protease 2 (TMPRSS2) for S protein to prime and then binds with SARS-CoV receptor angiotensin-converting enzyme 2 (ACE2) to enter the host cell [22], although there are many other reported receptors, such as CD147 and neuropilin-1 (NRP1) [23,24]. The combination of S protein and ACE2 receptor could result in a conformational change of S protein, which next leads to the cleaving of S1 and S2, thereby making the viral envelope protein enter through the endosomal pathway and fuse with the host cell membrane and release viral RNA [25]. After releasing viral RNA into the cytoplasm, the genomic RNA uses Hel and RdRp to start replicating and translating into structural proteins to assemble mature virions for release, leading to an immune response to SARS-CoV-2 infection [26] (see Figure 1).

## 3. Innate Immune Responses to SARS-CoV-2 Infection

The innate immune cells could initiate innate immune responses to virus infection after sensing virus-related molecular patterns via their PRRs. The initial host immune responses involve the activation of the type I and III IFN responses, followed by the release of pro-inflammatory cytokines and chemokines, thereby providing antiviral defense against viral infection [27]. Type I and type III IFNs bind to their receptors (IFNAR are IFNLR) and then activate interferon-stimulated genes (ISGs) [28]. Hundreds of ISGs are produced to exert antiviral effects by preventing viruses from entering, replicating and budding. By recruiting immune cells to the infected site and activating the adaptive immune response, the production of pro-inflammatory cytokines contributes to form the overall immune response. More and more evidences have shown that severe COVID-19 patients have a dysregulated immune response, leading to the development of viral hyperinflammation [29]. The elevated serum cytokines, especially interleukin-1 (IL-1), IL-6 and other pro-inflammatory cytokines, indicate that Toll-like receptors (TLRs)-mediated viral PAMPs could play a vital role in the pathogenesis of CoVs at the early stage [30]. The TLRs that can recognize the SARS-CoV-2 PAMPs may be present in dendritic cells (DCs) and alveolar macrophages [31].

Although multiple host cells produce a variety of cytokines and chemokines during virus infection, type I IFNs are the main cytokines involved in the antiviral response and are initiated through the recognitions of viral PAMPs by host PRRs such as TLRs, RIG-I-like receptors (RLRs) and NOD-like receptors (NLRs) [32,33,34]. TLRs and RLRs sense the viral genome and superficial proteins, which are found in tissue resident and migratory DCs, triggering inflammatory and chemokine responses. After SARS-CoV-2 infection, the retinoic-acid inducible gene I (RIG-I) or melanoma differentiation-associated gene 5 (MDA5) senses the genomic RNA stem-loop and/or double-stranded RNA (dsRNA) generated during viral replication, which leads to a conformational shift to expose the caspase activation recruitment domain (CARD) of RIG-I or MDA5. The exposure of CARD associates with the mitochondrial antiviral signaling protein (MAVS) and then convenes various downstream signaling components to mitochondria, containing an inhibitor of κ-B kinase ɛ (IKKɛ) and TANK-binding kinase 1 (TBK1) [35]. The activation of these kinases results in IFN regulatory factor (IRF) phosphorylation, resulting in nuclear translocation, and then stimulates the production of type I IFNs [36].

**Figure 2:**
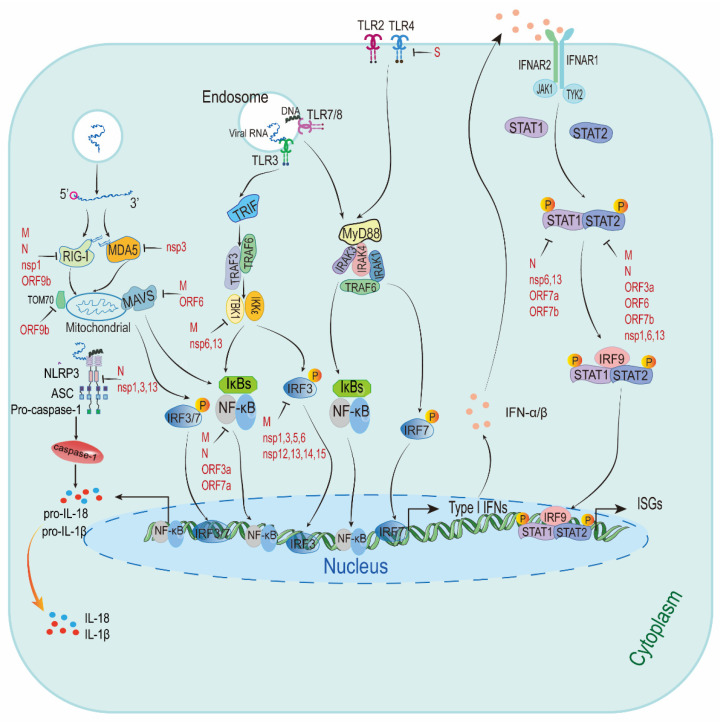
The predictive model describing the innate immune responses to SARS-CoV-2. There is a simplified figure of the type I IFNs responses after sensing SARS-CoV-2. It has been suggested SARS-CoV-2 escape type I IFNs signaling pathway and ISGs by targeting type I IFNs signaling. Viral proteins with type I IFNs inhibitory activities are highlighted in red. RIG-I: retinoic-acid inducible gene I; MDA5: melanoma differentiation-associated gene 5; MAVS: mitochondrial antiviral signaling protein; nsp1: non-structural protein; TOM70: translocases of outer membrane 70; NLRP3: NLR family pyrin domain-containing 3; ASC: apoptosis-associated speck-like protein containing a CARD; pro-IL-18: pro-interleukin (IL)-18; TLR3: Toll-like receptors 3; TRIF: TIR domain-containing adaptor inducing interferon-β; TRAF3: tumour necrosis factor receptor-associated factor 3; TBK1: TANK-binding kinase 1; IKKε: inhibitor of κ-B kinase ε; IκBs: inhibitor κB; NF-κB: nuclear factor κB; IRF3: IFN regulatory factor 3; MyD88: myeloid differentiation primary response 88; IRAK3: interleukin-1-receptor-associated kinase 3; IFNAR1: IFN receptor type I; JAK1: Janus-activated kinase 1; TYK2: tyrosine kinase 2; STAT1: signal transducer and activator of transcription 1; ISGs: interferon-stimulated genes.

Type I IFNs combine with IFNAR1/2 complexes on the cell surface, triggering the phosphorylation of preassociated Janus-activated kinase 1 (JAK1) and tyrosine kinase 2 (TYK2), which in turn phosphorylate the receptors at specific intracellular tyrosine residues. This leads to the recruitment and phosphorylation of signal transducer and activator of transcription 1 (STAT1) and STAT2 proteins [37]. The IRF9 interacts with phosphorylated STAT1 and STAT2 heterodimer to produce the IFN-stimulated gene factor 3 (ISGF3) complex. This complex binds to the IFN-stimulated response elements (ISREs) and activates their transcription to initiate the transcription of ISGs [38]. ISGs and other downstream constituents controlled by type I IFNs (including pro-inflammatory cytokines) could directly inhibit virus replication and recruit and activate various immune cells [39] (see Figure 2).

### 3.1. RLRs

RIG-I belongs to cytosolic RNA helicase family and can detect viral dsRNA [40]. Once activated, it triggers downstream pathways to produce type I IFNs and proinflammatory cytokines [41]. When co-transfected with plasmids expressing M protein and RIG-I or MDA-5 in HEK293T cells, type I and III IFNs were suppressed by SARS-CoV-2 M protein through reducing RIG-I/MDA-5 expression, which then weakened antiviral immunity and improved the replication of the virus [42]. In another study, it was shown that the overexpression of the SARS-CoV-2 N protein in HEK293T cells reduced both IFN-β and ISRE-dependent IFN signaling mediated by RIG-I activation in a dose-dependent manner using a luciferase reporter assay [43]. SARS-CoV-2 ORF9b targets the nuclear factor κB (NF-κB) essential modulator (NEMO or IKKγ) and stops K63-linked polyubiquitination to inhibit the classic IκB kinase α (IKKα)/β/γ-NF-κB signaling transduction and subsequently IFN production in HEK293T cells [44]. Nsp1 is capable of suppressing the induction of RIG-I and ISG15 upon IFN-β stimulation on the protein level but not the mRNA level [45]. In summary, these evidences suggest that inhibiting RIG-I activation by the above targets provides a strategy for innate immune evasion by SARS-CoV-2 (see Table 1).

MDA5 is a member of the RLR family; its structure is very similar to RIG-I and can sense intracellular dsRNA [65]. MDA5 contains two CARDs near its N terminus and a DExD/H-box helicase domain. After MDA5 binds to the viral ligand in the C-terminal domain, MDA5 promotes conformational changes that allows CARD to bind to MAVS, finally leading to producing type I IFNs and pro-inflammatory cytokines through the activation and nuclear translocation of IRF3 and NF-κB [66]. The PLpro (the protease domain of nsp3) of SARS-CoV-2 was confirmed to interact with MDA5 and antagonize ISG15-dependent MDA5 activation through direct de-ISGylation [59]. As a result, inhibiting MDA5 activation provides a new strategy for SARS-CoV-2 to evade the host’s antiviral innate immunity (see Table 1).

### 3.2. MAVS

MAVS is a pivotal adapter in cellular antiviral innate immunity [40]. The MAVS CARD binds to similar CARDs present in RIG-I and MDA5, which induces MAVS activation [67]. SARS-CoV-2 M protein directly targets MAVS to inhibit the innate immune response [50]. ORF6 also affects IFN production. When transfecting HEK293T cells with a plasmid expressing ORF6, RIG-I, MDA5, MAVs and an IFN-β reporter plasmid, the overexpression of ORF6 inhibited RIG-I, MDA5 and IFN promoter activation induced by MAVS [48]. Further studies are required to reveal the exact mechanisms of IFN and MAVS signaling suppression during SARS-CoV-2 infection (see Table 1).

### 3.3. TLRs

TLRs are involved in the developing and activating of innate immunity, containing 11 transmembrane receptor proteins which can recognize PAMPs. TLR4 is involved in the response, which is manipulated by oxidized phospholipids (OxPLs) induced by SARS-CoV-2 infection. TLR4 activation leads to the production of type I IFNs and inflammatory cytokines [e.g., IL-6 and tumor necrosis factor-α (TNF-α)] through myeloid differentiation primary response 88 (MyD88) and TIR domain-containing adaptor inducing interferon-β (TRIF) [68]. TLR3 and TLR7/TLR8 sense viral RNA in the endosome and activate IRF3/IRF7 to produce type I IFNs through MyD88/TRIF signaling [69]. It has been found that the SARS-CoV-2 S protein is capable of interacting with and activating TLR4 in THP-1 cells [70]. Hence, improving excessive TLR4-mediated innate signaling may be beneficial to the treatment of COVID-19. In addition, Naltrexone, an approved opioid antagonist, is being repurposed for COVID-19 as TLR4 antagonist (see Table 1).

### 3.4. NLRP3

The activation of NLRP3 (NLR family pyrin domain-containing 3) is involved in the antiviral immune response and inflammatory reaction [71]. The NLRP3 inflammasome promotes inflammation by cleaving and activating key inflammatory molecules including active caspase-1 (Casp1p20), IL-1β and IL-18 [72]. The rapid transmission of SARS-CoV-2 is due to the inability of type I IFN responses to eliminate its infection, and this inability relates to SARS-CoV-2 virulence factors that influence rapid infectivity. It has been suggested that the SARS-CoV-2 unique domain (SUD) of nsp3 is involved in the virulence and pathogenesis by activating the signaling responses of the NLRP3 inflammasome, which are mainly responsible for the expression of CXCL10 and the secretion of IL-1β and trigger the infiltration of macrophages/monocytes in lung tissues [61]. Another study revealed that nsp1 and nsp13 inhibit NLRP3 inflammasome activation in THP-1 and HEK293T cells [40,57]. As reported [53], SARS-CoV-2 N protein directly connects with NLRP3 to assemble and activate NLRP3 inflammasome, which then leads to inflammatory cytokine production and lung injury in mice. Together, inhibiting NLRP3 inflammasome could bring down the “cytokine storm” and lung injury during SARS-CoV-2 infection, indicating NLRP3 inflammasome could be a target for COVID-19 treatment (see Table 1).

### 3.5. IRF

It is essential to express IRF-mediated type I IFNs and IFN-induced genes in responses to viral infection [73]. A co-immunoprecipitation experiment showed that ORF6 interacted with karyopherin α 2 (KPNA2) to block IRF3 nuclear translocation and subsequently inhibited IFN-β production [46]. In HEK293T cells, the N protein significantly inhibited the phosphorylated IRF3 induced by Sendai virus (SeV) infection or poly (I:C) (a dsRNA mimic) but did not inhibit the production of IRF3 [54]; however, there was a study that showed that SARS-CoV-2 nsp12 could not antagonize IFN-β [74]. Thus, more studies are needed to further clarify the IFN signaling mechanisms and IRF signaling inhibition during SARS-CoV-2 infection. SARS-CoV-2 M binds with TBK1 and then degrades TBK1 by the ubiquitin pathway, which finally inhibits IRF3 phosphorylation and suppresses type I IFNs production [51]. Nsp6 and nsp13 bind to TBK1, and then suppress the phosphorylation of IRF3 and TBK1 in HEK293T cells [46]. Interestingly, in another study, it was found that nsp13, nsp14 and nsp15 could inhibit IRF3 nuclear localization [64]. Many studies indicated that nsp1 prevents IFN induction in part by blocking IRF3 phosphorylation [58]. The protease domain of SARS-CoV-2 nsp3 (PLpro, the papain-like protease) also attenuates type I IFNs responses by cleaving IRF3 [60]. Another study revealed a new mechanism by which nsp5 antagonizes IFN production through retaining phosphorylating IRF3 in the cytoplasm [62]. It is not clear what the functions of IRF5 and IRF7 are and how they are activated through ubiquitinating or phosphorylating during SARS-CoV-2 infection. In addition, it is not known whether IRF5 and IRF7 participate in the regulation of the transcription of excessive proinflammatory cytokines in response to SARS-CoV-2 infection. In any case, this evidence suggests that inhibiting IRF activation may be an effective mechanism for SARS-CoV-2 immune evasion (see Table 1).

### 3.6. JAK/STAT Signaling

The engagement of type I IFNs and type II IFNs (IFN-γ) with their specific receptors results in autophosphorylating and activating JAK/ STAT pathways, which in turn regulates type I IFNs transcription [75]. SARS-CoV-2 N protein attenuated type I IFNs signaling accompanied by the decrease in p-STAT1 and p-STAT2 in HEK293T cells transfected by N protein [56]. Another study revealed that nsp1, 6, 13, ORF3a, 7b and M suppressed STAT1 phosphorylation while nsp6, 13, ORF7a and 7b inhibited STAT2 phosphorylation. ORF6 could also block STAT1 nuclear translocation [46]. More studies are needed to reveal the exact mechanism of type I IFNs inhibition during SARS-CoV-2 infection (see Table 1).

### 3.7. NF-κB

NF-κB is a family of transcription factors involved in host immunity and inflammation, which plays a crucial role in the host’s defense against viral infection [76]. The main mechanism for the activation of the NF-κB pathway is the phosphorylation of IκBα activated by the IKK complex, which consists of two catalytic subunits (IKKα and IKKβ) and a non-catalytic subunit NEMO/IKKγ. IKK can be induced by cytokines, microbial components and infectious factors, etc. [47]. The activated NF-κB pathway induces the abnormal expression of proinflammatory factors, which may be the reason for COVID-19 inflammation. The damaged human DNA at later stages of COVID-19 could overactivate STING (stimulator of IFN genes), leading to producing IFN-β by activating interferon regulator factor 3 (IRF 3) [77]. It has been proposed that the SARS-CoV-2 N protein undergoes liquid–liquid phase separation (LLPS) by binding to viral RNA and forms functional membrane-less organelles to recruit TAK1 and IKK complex, the key kinases of NF-κB signaling, thereby promoting NF-κB activation [55]. Using a reporter assay, it was found that SARS-CoV-2 ORF3a, 7a, M and N could activate NF-κB and promote p65 nuclear localization [47]. Together, NF-κB inhibitors may be effective antiviral drugs against COVID-19 (see Table 1).

### 3.8. TOM70

Translocases of outer membrane 70 (TOM70), acting as a surface receptor of the translocase of the outer membrane complex, is the gateway for mitochondrial protein import [78], and it is an important component in the signal cascade that results in innate immune activation [79]. ORF9b, a SARS-CoV-2’s particular accessory protein, participates in immune evasion through targeting mitochondria, where it correlates with the multifunctional adapter TOM70. A previous study revealed that SARS-CoV-2 ORF9b localized on mitochondria and reduced the activation of type I IFNs in HEK293T cells transfected with HA-TOM70_∆TM_ alone or along with ORF9b-Flag [49]. There may be two possible explanations for how ORF9b suppresses type I IFNs responses by interacting with TOM70. One, the chaperone heat shock protein (Hsp) 90 associates with TOM70 and plays a key role in the TOM70-mediated type I IFNs activation, and ORF9b may compete with HSP90 [80]. Two, TOM70 may be critical to mitochondrial energy metabolism, and ORF9b may induce the production of lactic acid that was proven to inhibit type I IFNs responses through interacting with TOM70 [81]. Based on the above evidence, targeting ORF9b-TOM70 interaction may be a novel therapeutic tool for COVID-19 (see Table 1).

### 3.9. TBK1

TBK1 belongs to the IKK-kinase family of kinases of the innate immunity signaling pathways [82], and it is important for IFN-β production and congenital antiviral immunity [83]. It had been reported that the SARS-CoV-2 M protein could inhibit TBK1-induced IFN-β-driven luciferase reporter plasmid (IFN-β-Luc) in a dose-dependent manner. The phosphorylated IRF3 caused by TBK1 was reduced in cells transfected with SARS-CoV-2 M, which provided evidence for the inhibitory effect of M on TBK1-induced innate immunity [51]. Furthermore, nsp6-, nsp13- and TBK1-binding suppressed phosphorylated IRF3 and TBK1, respectively, thereby reduced IFN-β production [46]. These studies suggest that TBK1 is a potential target for developing effective treatment strategies (see Table 1).

## 4. Conclusions and Perspectives

SARS-CoV-2 is affecting the health and safety of the global community at present, and the pandemic of its disease COVID-19 is considered the worst global health crisis since the Spanish flu of 1918. In such difficult circumstances, rapid mobilization by health authorities and the medical community, as well as the strength of the research community, is required to find the best diagnostic methods, prevention and treatment.

COVID-19 features as reduced innate antiviral defenses and exuberant inflammatory cytokine production [84]. One major component of the pathogenesis of COVID-19 is the dysregulation of innate immune responses, and here we found that SARS-CoV-2 seems well-adapted to avoid and suppress the type I IFNs response through the interplay between viral proteins (such as M and N) and host innate receptors or regulators (e.g., RIG-I and MDA5). Such effective strategies help SARS-CoV-2 to replicate and transmit in infected individuals without meeting with the host defense. IFN-α/β therapy is expected to enhance the antiviral response of COVID-19 infections at the early stage and, if possible, at the site of infection. In fact, a clinical trial showed that IFN-β therapy appeared to be critical to improving patient outcomes with a joint therapy of IFN-β, lopinavir–ritonavir and ribavirin [85]. Even so, the excessive release of pro-inflammatory cytokines observed in the late period of COVID-19 infection may affect the efficiency of IFN-α/β treatment after the onset of symptoms. The adverse reactions of improper, excessive or untimely type I IFN responses in viral infections have indeed attracted people’s attention [86]. Therefore, it is necessary to weigh the benefits, risks and the best time window for IFN administration [87,88].

The current study aims to better understand the molecular and cellular signaling mechanisms underlying SARS-CoV-2 infection and host–virus interactions. Finding the answers to these questions is not only crucial for a better understanding of basic knowledge of SARS-CoV-2 but is also helpful for developing antiviral therapeutics against the COVID-19 pandemic.

## Figures and Tables

**Table 1:** Summary of targets of SARS-CoV-2 proteins or pathways.

Viral Proteins	Host Proteins or Pathways Targeted	Cellular Model	References
ORF3a ORF6 ORF7a ORF7b ORF9b M	(a) the phosphorylation of STAT1 (b) NF-κB (a) RIG-I/IFN-β; MDA5/IFN-β; MAVS/IFN-β (b) IRF3/type I IFNs response; the nuclear translocation of STAT1 (a) the phosphorylation of STAT2 (b) NF-κB the phosphorylation of STAT1 and STAT2 (a) RIG-I-MAVS (b) TOM70 (a) RIG-I/MDA-5 (b)MAVS (c) IRF3, TBK1 (d) the phosphorylation of STAT1 (e) NF-κB	(a) HEK293T, Vero, BHK-21 and Huh-7 cells(b) HeLa, A549 and 16HBE14o cells (a) Human 293T, 293, Calu-3, HeLa and Vero cells (b) HEK293T, Vero, BHK-21, Huh-7 cells (a) HEK293T, Vero, BHK-21, Huh-7 cells (b) HeLa, A549 and 16HBE14o cells HEK293T, Vero, BHK-21, Huh-7 cells HEK293T cells (a) HEK293, HEK293T, HeLa, Vero cells (b) HEK293, HeLa and HEK293T cells (c) HEK293T cells (d) HEK293T, Vero, BHK-21, Huh-7 cells (e) HeLa, A549 and 16HBE14o cells	[42,44,46,47,48,49,50,51]
S	TLR4/TRIF/IFN-β	ACE2-positive type II alveolar cells in the lungs	[52]
N	(a) RIG-I (b) NLRP3(c)IRF3 (d)NF-κB (e)the phosphorylation of STAT1 and STAT2.	(a) A549, HeLa, and HEK293T cells (b) HEK293T, HeLa, and A549 cells (c)Vero E6, HEK293T, HeLa, A549, Huh7 cells(d)HEK293T cells	[43,53,54,55,56]
nsp1 nsp3 nsp3c nsp5	(a) RIG-I/ IFN-β (b) NLRP3 (c) IRF3 (d) the phosphorylation of STAT1 (a) MDA5 (b) IRF3 NLRP3 IRF3	(a) HEK293T cells (b) HEK293T cells (c) A549, Vero E6, HEK 293T, Huh7 cells (d) HEK293T, Vero, BHK-21, Huh-7 cells (a) HEK293T, HEK293, HeLa, MEF, NHLF, Vero, A549–hACE2, BHK-21 cells (b) A549 and HeLa cells A549 cells HEK293, HeLa and A549 cells	[45,46,57,58,59,60,61,62]
nsp6 nsp12	(a) IRF3; the phosphorylation of STAT1 and STAT2 (b) TBK1 IRF3	(a) HEK293T, Vero, BHK-21, Huh-7 cells (b) HEK293T, Vero, BHK-21, Huh-7 cells HEK293T, HeLa, and Vero cells	[46,63]
nsp13 nsp14	(a) NLRP3(b) the phosphorylation of STAT1 and STAT2 (c) IRF3 (d) TBK1 IRF3	(a) HEK293T cells (b) HEK293T, Vero, BHK-21, Huh-7 cells (c) HEK293T cells (d) HEK293T, Vero, BHK-21, Huh-7 cells HEK293T cells	[46,57,64]
nsp15	IRF3	HEK293T cells	[64]

## Data Availability

Data is contained within the article, which can be found in the cited publications.
